# Mechanism of endothelial progenitor cell recruitment into neo-vessels in adjacent non-tumor tissues in hepatocellular carcinoma

**DOI:** 10.1186/1471-2407-10-435

**Published:** 2010-08-17

**Authors:** De-cai Yu, Jun Chen, Xi-tai Sun, Lin-yuan Zhuang, Chun-ping Jiang, Yi-tao Ding

**Affiliations:** 1 Institute of Hepatobiliary Surgery, Affiliated Drum Tower Hospital, School of Medicine, Nanjing University, Nanjing, Jiangsu Province, China; 2 Department of Hepatobiliary Surgery, Affiliated Drum Tower Hospital, School of Medicine, Nanjing University, Nanjing, Jiangsu Province, China; 3 Department of Pathology, Affiliated Drum Tower Hospital, School of Medicine, Nanjing University, Nanjing, Jiangsu Province, China

## Abstract

**Background:**

We investigated the distribution and clinical significance of mobilized endothelial progenitor cells (EPCs) in hepatocellular carcinoma (HCC). We found that many more EPCs were recruited to nonmalignant liver tissue (especially into adjacent non-tumor tissues (AT)) than to tumor vessels. These results suggest that the mechanism underlying the recruitment of EPCs into microvessels in AT merits further investigation

**Methods:**

Angiogenic factors were detected in three tissue microarrays comprising normal liver, paired tumor tissue (TT) and AT from 105 patients (who had undergone hepatectomy for HCC) using immunohistochemistry. Also, the number of EPCs (positive for Sca-1, Flk-1 and c-Kit) in the blood and liver of cirrhotic mice were determined by flow cytometry and immunohistochemistry. The distribution of these labeled EPCs in tumor and non-tumor tissues was then studied.

**Results:**

The results from the tissue microarrays showed that the expression levels of VEGF-A, bFGF, TGF-β, MCP-1, TSP-1, MMP-9, TIMP-2, and endostatin were significantly higher in AT than in either normal liver or TT (*p *< 0.05), but no significant difference was found in the expression levels of COX-2 and NOS-2 between AT and TT. The expression of VEGF-A, bFGF, TGF-β, MCP-1, TSP-1, MMP-9, TIMP-2, endostatin, COX-2, and NOS-2 in normal liver tissue was weaker than that in AT or TT. In cirrhotic mice, the number of circulating endothelial progenitor cells gradually increased, before decreasing again. In this mouse model, increased numbers of EPCs were recruited and homed specifically to the cirrhotic liver.

**Conclusions:**

Both liver cirrhosis and HCC led to increased expression of pro-angiogenic factors, which resulted in the recruitment of EPCs into AT. Also, EPCs were mobilized, recruited and homed to cirrhotic liver. The unique pathology of HCC coupled with liver cirrhosis may, therefore, be associated with the distribution and function of EPCs.

## Background

It is generally accepted that tumors are endowed with angiogenic capabilities, and that their growth, invasion and metastasis are angiogenesis-dependent. Over the past several years, a substantial amount of evidence from both animal and human studies suggests that neoangiogenesis involves endothelial progenitor cells (EPCs) as well as endothelial cells (ECs) co-opted from the surrounding vessels [1-3]. Some studies have reported that EPCs are mobilized, recruited and home with high specificity to solid tumors [4,5]. Therefore, EPCs might be useful for the early detection of tumors, for distinguishing benign from malignant disease, for determining prognoses, predicting the response to therapy, and monitoring the clinical course [6-8]. However, our previous studies of hepatocellular carcinoma (HCC) suggest that many more EPCs are recruited into non-malignant liver tissue, especially into adjacent non-tumor tissues (AT), than are recruited into tumor vessels [9]. The mechanism underlying this increased recruitment into microvessels in AT is unknown.

The recruitment and homing of EPCs is affected by hypoxia, angiogenic factors, and adhesion molecules [10]. These factors are also relevant to HCC with liver cirrhosis [11-13]. HCC is associated, in most cases, with chronic liver diseases including chronic viral hepatitis and cirrhosis, especially in Southeast Asia. The pathology of HCC with liver cirrhosis may be an important factor in the distribution, contribution, and differentiation of EPCs.

Therefore, in this study, the distribution and expression levels of angiogenic factors related to the mobilization and recruitment of EPCs were evaluated using HCC tissue microarrays. Also, using a mouse model of liver cirrhosis, the number of endothelial progenitor cells in the circulation and tissues was also determined to evaluate their mobilization, recruitment, and distribution characteristics.

## Methods

### Tissue arrays

Patients and tissue samples: a group of 105 patients with HCC received curative resection in the Department of Hepatobiliary Surgery in Nanjing Drum Tower Hospital, Medical College of Nanjing University. The mean age of these patients was 50.0 years (50.0 ± 12.5 years). In 72 of these patients (68.6%), the disease was associated with liver cirrhosis, and 85 patients (81.0%) were positive for serum hepatitis B surface antigen. The mean size of the resected HCC in this study was 7.3 cm. Sixty-six patients had a large tumor (maximum diameter > 5 cm), 39 had a small tumor (maximum diameter ≤5 cm), and 48 had multiple tumors (more than two nodules). Before surgery, no chemotherapy or other treatment was given and none of the patients had extra-hepatic metastasis. In addition, 44 liver tissue samples without evidence of HCC or liver cirrhosis were used as negative controls.

A tissue microarray was constructed as previously described [14]. Briefly, surgical specimens were fixed in 10% formalin and embedded in paraffin. In accordance with the Classification of Carcinomas of the Liver (Modified) proposed by UICC, sections (4 μm thick) were cut from the blocks and stained with hematoxylin and eosin (H&E) to study the pathological features of HCC [15]. Representative regions of the blocks were identified on the H&E-stained sections and cylindrical tissue cores (1 mm in diameter) were prepared using a tissue microarray constructor (Beecher Instruments MTA-1, Silver Spring, MD, USA). Two cores (paired tumor tissue (TT) and AT) from each donor block were inserted into a pre-formed recipient block to produce a tissue array block. Thereafter, 4 μm thick consecutive sections were cut from the array block for immunohistochemical staining.

### Immunohistochemical staining

Using the tissue array, the expression and distribution of 17 angiogenic biomarkers were evaluated immunohistochemically. These included the165-amino acid isoform of vascular endothelial growth factor (VEGF-A), basic fibroblast growth factor (bFGF; Santa Cruz), matrix metalloproteinase-9 (MMP-9), endostatin, tissue inhibitor of metalloproteinases-1 and -2 (TIMP-1, TIMP-2), thrombospondin-1 (TSP-1; (Neomarker), p53, transforming growth factor-β (TGF-β), cyclooxygenase 2 (COX-2; DAKO), and monocyte chemotactic protein 1 (MCP-1; Chemicon). Briefly, tissue sections were de-waxed in xylene, dehydrated in ethanol, and then treated with 3% hydrogen peroxidase for 30 min to block endogenous peroxidase activity. After incubation with 10% normal goat serum for 20 min to reduce non-specific binding, the sections were incubated with primary antibodies over night at 4°C. The sections were then incubated with the corresponding secondary antibody for 30 min followed by visualization using 3, 3'-diaminobenzidine. An immunoglobulin-negative control was used to rule out nonspecific binding.

Immunohistochemical staining was assessed by two independent investigators without any knowledge of the corresponding clinicopathological data. Positive cells were counted in representative areas of the sections and expressed as a percentage. The level of immunostaining was categorized into three grades: - = negative; 5%-50% = low; >50% = moderate-high, as reported by Au NH [16]

### EPCs labeled with CM-Dil

Mouse bone marrow derived EPCs were induced and cultured using the EGM-2 MV Bullet Kit (CC-3202, Clonetics/BioWhittaker) as in our previous study [9]. After passaging three times, EPCs were digested with 0.05% trypsin and resuspended in PBS. EPCs were labeled with CM-Dil (Molecular Probes Inc., USA) according to the method of Ferrari [17]. CM-Dil was dissolved in DMSO (final concentration of 1 mg/ml). EPCs (10^6 ^cells) were incubated in 2 μL CM-Dil at 37°C for 5 min, followed by 4°C for 15 min, washed twice with PBS and suspended in EGM-2 medium. The percentage EPCs positive for CM-Dil was evaluated by flow cytometry.

### Animal model of liver cirrhosis

This study used 8-week-old Kunming male mice weighing 20 grams. Liver cirrhosis was induced by subcutaneous injection of 10% CCI_4 _(100 μL) every third week for three months and drinking water containing 8% alcohol. Animal care and experimental procedures were approved by the research ethics committee of Drum Tower Hospital.

### EPC transplantation

Labeled EPCs (10^6 ^cells) were infused into cirrhotic mice via the tail vein. After 48 hours, the liver, lungs, spleen, and kidneys were excised, embedded in OCT and stored in liquid nitrogen. Tissue slices (4 μm thick) were observed using a fluorescence microscope.

### Flow cytometry

EPCs in the circulating unselected peripheral blood cell population from cirrhotic mice were measured by flow cytometry at 0, 2, 4, 8 and 12 weeks. EDTA-anti-coagulated blood (100 μL) was incubated for 30 min at 4°C with 5 μL phycoerythrin-conjugated anti-Flk-1 (Miltenyi Biotec, Germany), PerCP-conjugated anti-c-Kit, or FITC-conjugated anti-Sca-1 (Beckton Dickinson, Heidelberg, Germany). IgG1-FITC and IgG2α-phycoerythrin antibodies (B&D) served as isotype controls for each procedure. After incubation, cells were lysed and washed with PBS before analysis. A minimum of 50,000 events were acquired for each sample. Quantitative fluorescence analysis was done using FACSCalibur and WinMDI software (B&D).

## Results

### Higher expression of angiogenic factors in adjacent non-tumor tissues than in tumor tissues

The mobilization, recruitment and homing of EPCs to tumor tissues are multi-step events requiring the participation of multiple factors, including angiogenic factors, adhesion molecules, tumor cells, ECs, and stromal cells [10,18]. Our previous studies showed higher expression of VEGF, HIF-1α, CD105 and AC133 in AT [9,19]. Therefore, we hypothesized that AT might be hypoxic and an area of active angiogenesis. To confirm this, we evaluated the expression of some key angiogenic factors, including activator molecules (VEGF-A, bFGF, TGF-β, MCP-1, MMP-9, TSP-1), inhibitor molecules (endostatin, TIMP-1, TIMP-2), and the transcript factors (COX-2, NOS-2), in AT and TT using HCC tissue arrays.

**Figure 1:**
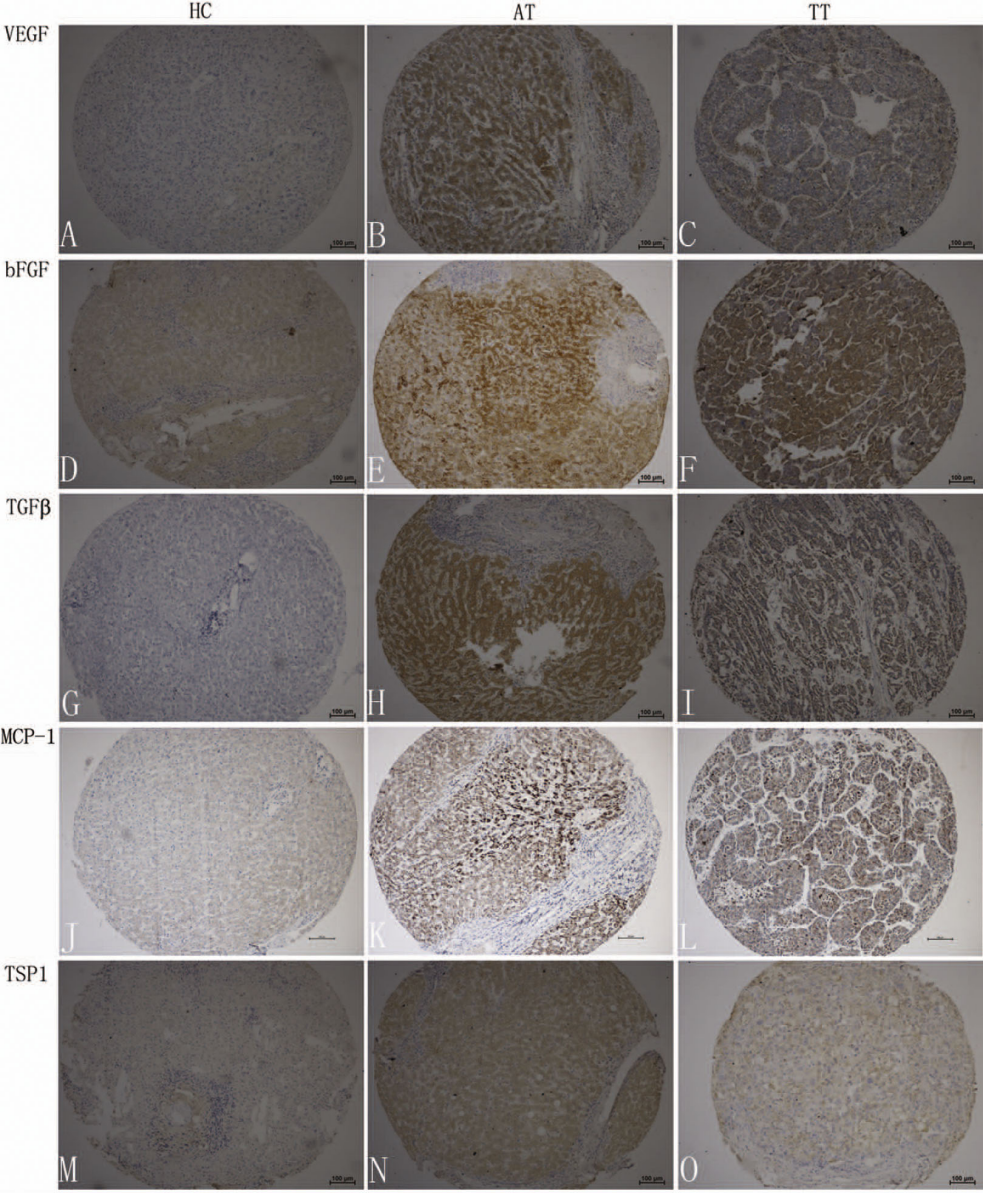
**Immunohistochemical staining of VEGF-A, bFGF, TGF-β, MCP-1, TSP-1 in tissues**. Representative sections showing immunohistochemical staining of VEGF-A (A, B, and C), bFGF (D, E, and F), TGF-β (G, H, and I), MCP-1 (J, K, and L), TSP-1 (M, N, and O) in healthy controls (A, D, G, J, and M), adjacent non-tumor tissues (B, E, H, K, and N) and tumor tissues (C, F, I, L, and O). The signals were detected by DAB staining.

**Figure 2:**
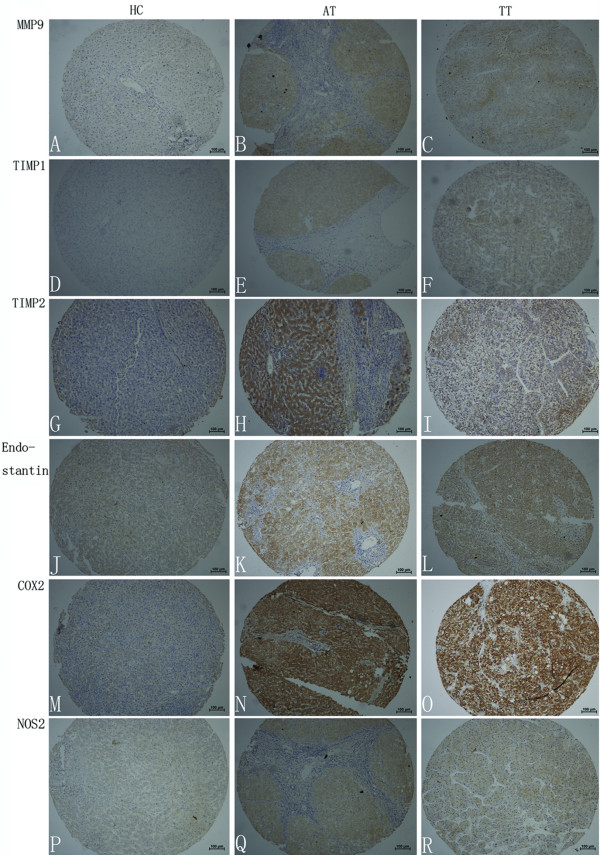
**Immunohistochemical staining of MMP-9, TIMP-1, TIMP-2, endostatin, COX-2, and NOS-2 in tissues**. Representative sections showing immunohistochemical staining of MMP-9 (A, B, and C), TIMP-1 (D, E, and F), TIMP-2 (G, H, and I), endostatin (J, K, and L), COX-2 (M, N, and O), and NOS-2 (P, Q, and R) in healthy controls (A, D, G, J, M, and P), adjacent non-tumor tissues (B, E, H, K, N, and Q) and tumor tissues (C, F, I, L, O, and R). The signals were detected by DAB staining.

Immunoreactivity to VEGF-A, bFGF, TGF-β, MCP-1, TSP-1, TIMP-1, TIMP-2, and endostatin was observed mainly in HCC cells and hepatocytes, showing a predominant cytoplasmic staining with positive liver cells distributed throughout the tumor tissue and the surrounding liver (Figures 1 and 2). Cytoplasmic and nuclear staining for COX-2 and NOS-2 was observed in both tumor cells and non-tumor connective tissue cells, including sinusoidal endothelial cells.

The expression of VEGF-A, bFGF, TGF-β, MCP-1, MMP-9, TSP-1, TIMP-2, and endostatin was significantly higher in AT than in normal liver or TT (*p *< 0.01 and 0.05, respectively), while no significant difference was found in the levels of TIMP-1, COX-2, and NOS-2 between TT and AT. Meanwhile, constitutive expression of VEGF-A, bFGF, TGF-β, MCP-1, TSP-1, MMP-9, TIMP-2, and endostatin in normal liver tissue was weaker than that in AT or TT tissues (Table 1, Figures 1 and 2). This suggests that AT is hypoxic and is an area of active angiogenesis, resulting in increased recruitment of EPCs.

**Table 1:** Expression and distribution of angiogenic molecules in HCC tissue arrays

	HC				AT				HCC			
			
	(-)	(+)	(++)	Positive Percent	(-)	(+)	(++)	Positive Percent	(-)	(+)	(++)	Positive Percent
VEGF-A	35	3	6	20.5%	43	9	46	56.1%**	41	29	27	57.7%^^^##^
bFGF	27	4	13	38.6%	29	13	55	70.1%**	32	41	23	66.7%^^^##^
TGF-β	20	8	16	54.5%	11	4	83	88.8%**	14	15	68	85.6%^^^#^
MCP-1	20	8	8	44.4%	32	21	44	67.0%*	59	21	17	39.2%^##^
TSP-1	38	4	1	11.6%	43	14	41	56.1%**	37	33	27	61.9%^^^##^
MMP-9	28	7	9	36.4%	25	13	59	74.2%**	40	23	35	59.2%^^##^
TIMP-1	24	2	13	38.5%	63	14	17	33.0%	69	10	17	28.1%
TIMP-2	21	6	17	52.3%	19	13	66	80.6%**	26	37	35	73.5%^^^##^
Endostatin	23	9	10	45.2%	14	9	75	85.7%**	37	21	40	62.2%^##^
COX-2	25	9	10	43.2%	7	39	51	92.8%**	9	46	43	90.8%^^
NOS-2	32	9	3	27.3%	37	21	40	62.2%**	43	30	25	56.1%^^

*p < 0.05, **p < 0.01, AT versus HC; ^#^*p *< 0.05, ^##^*p *< 0.01, HCC versus AT, ^*p *< 0.05; ^^*p *< 0.01, HCC versus HC

### Bone marrow EPCs are mobilized and recruited into cirrhotic liver

In general, EPCs are recruited into tumor tissues [4-8]. However, in HCC many more EPCs are recruited into AT than into TT [9]. In Southeast Asia, 80% of patients with HCC also have hepatitis or liver cirrhosis. Therefore, the particular pathology of HCC with concomitant liver cirrhosis may play an important role in the distribution of EPCs.

**Figure 3:**
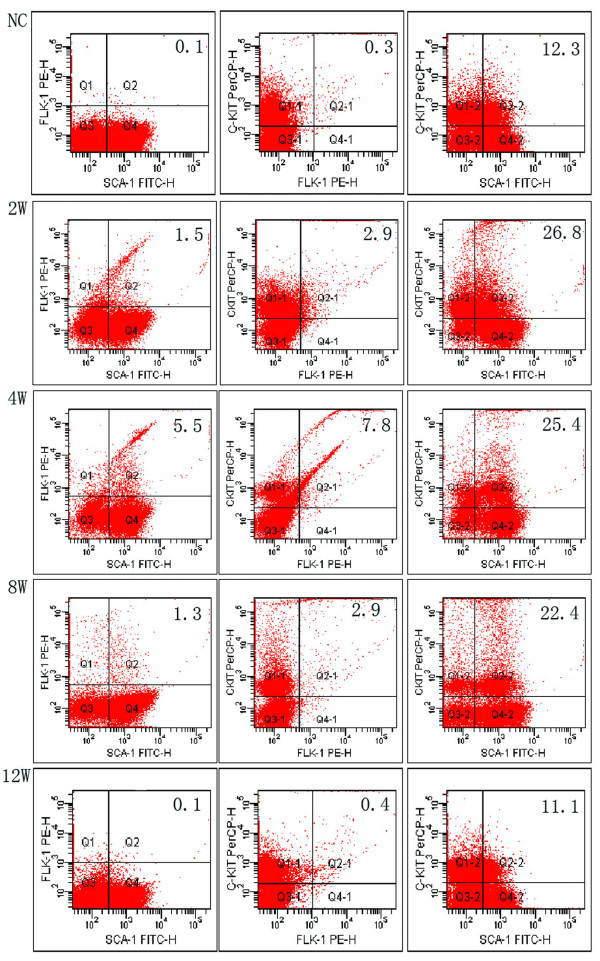
**Dynamic changes in the levels of circulating EPCs during liver cirrhosis**. The mean percentage of Sca-1, c-Kit and Flk-1 positive cells is shown (*n *= 5) during liver cirrhosis (0, 2, 4, 8 and 12 weeks post disease onset).

**Figure 4:**
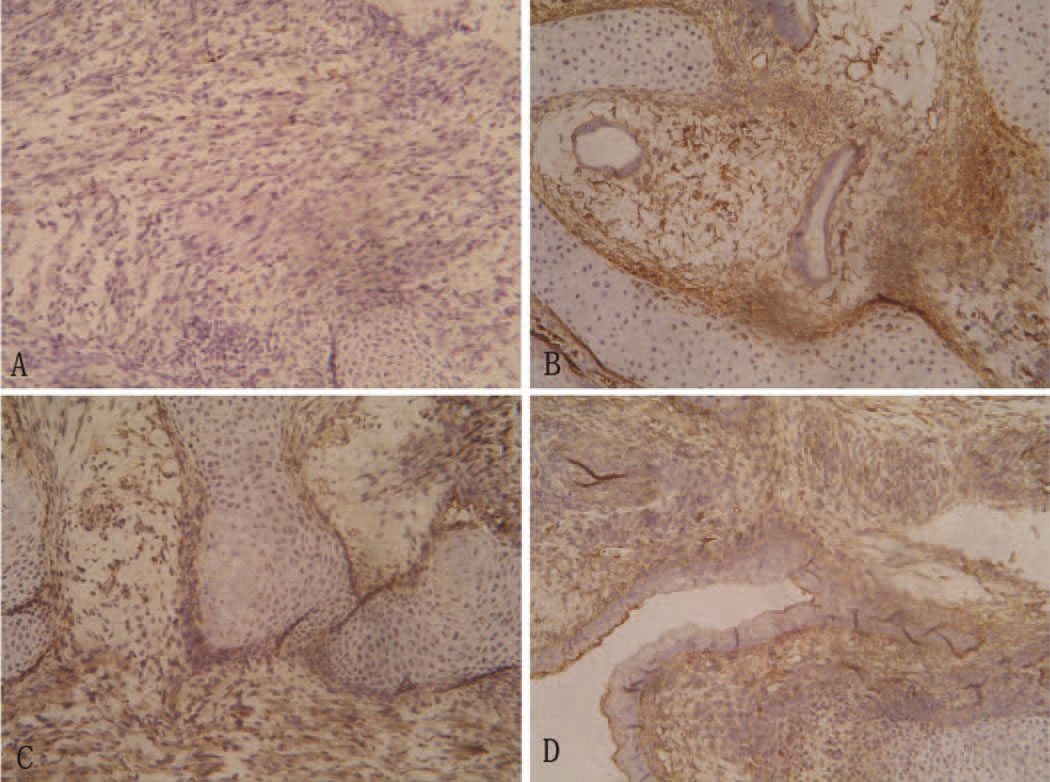
**Distribution of Sca-1 positive cells in cirrhotic liver during cirrhosis**. Representative sections showing distribution of Sca-1 positive cells in cirrhotic liver at 0 week (control, A), and at 2 weeks (B), 4 weeks (C), and 8 weeks (D). The signals were detected by DAB staining. Magnification: ×200.

Sca-1, Flk-1, and c-Kit are surface markers of mouse EPCs [20,21]. EPCs in the circulation and livers of cirrhotic mice were analyzed by flow cytometry and immunohistochemical staining. The number of circulating EPCs gradually increased up to 4 weeks after disease onset and then deceased gradually (Figure. 3 and Additional file 1). Moreover, consistent with the distribution of AC133-positive cells found in our previous study of human HCC with cirrhosis, increasing numbers of Sca-1 positive cells were observed in the cirrhotic livers, especially in the portal areas (Figure. 4) [9].

### EPCs are recruited and home with high specificity to cirrhotic liver

**Figure 5:**
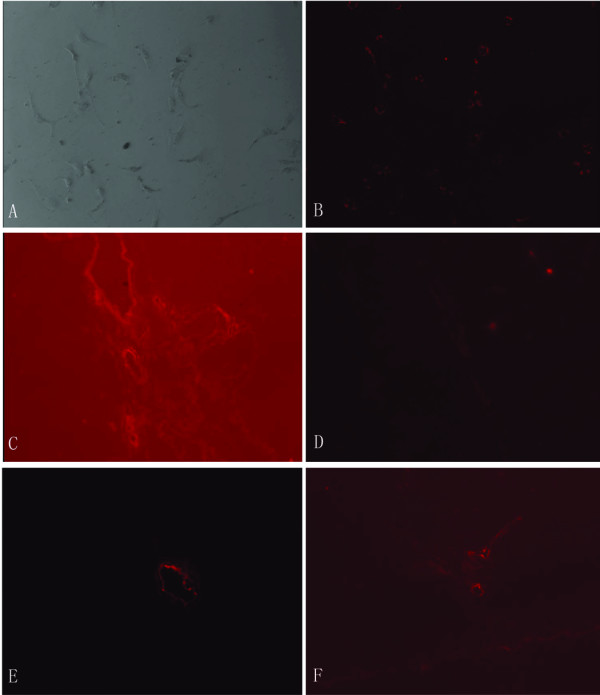
**Biodistribution of CM-Dil-labeled-EPCs in the cirrhotic mouse**. Representative sections showing biodistribution of CM-Dil-labeled-EPCs (A, B) in liver (C), lung (D), kidney (E) and spleen (F). Magnification: ×40.

EPCs labeled with CM-Dil were used to evaluate the bio-distribution of EPCs in mice with liver cirrhosis. Flow cytometry showed that the majority (94.5%) of EPCs were labeled with CM-Dil (Additional file 2). Approximately 10^6 ^CM-Dil-labeled EPCs were infused into the tail vein and the liver, lungs, spleen, and kidneys were excised 48 hours later and embedded in OCT. Fluorescence microscopy showed high numbers of labeled EPCs in the portal areas of the cirrhotic livers, but few EPCs in the lungs, spleen, or kidneys (Figure 5). This clearly indicates that EPCs were recruited and homed with high specificity to the cirrhotic liver.

## Discussion

Neoangiogenesis involves EPCs as well as ECs co-opted from surrounding blood vessels. The mobilization, recruitment, homing and incorporation of EPCs into tumors are independent multi-step events in tumor vasculogenesis. This complex process requires the participation of multiple growth factors, tumor cells, ECs, stromal cells and EPCs in the tumor microenvironment [18,22,23]. Pro-angiogenic factors such as the VEGF-A, bFGF, and MCP-1 are involved in the activation, mobilization and recruitment of EPCs from the bone marrow and in promoting the differentiation of EPCs into ECs in some ischemic diseases and during tumor growth [24,25]. Studies have found that there is higher expression of many angiogenic factors and increased recruitment of EPCs into non-tumor tissues compared with tumors. This is supported by this current study and our previous study [9,19]. Furthermore, these factors activate MMPs, particularly MMP-9, leading to the release of soluble Kit ligand which, in turn, promotes cell proliferation and motility within the bone marrow [17,26]. Also, the expression of adhesion molecules that enable the homing of EPCs is higher in AT (Additional file 3 and 4).

Because ECs in the liver release a battery of different CC chemokines, it is possible that these CC chemokines play a role in EPC homing to HCC liver tissues. It is worth noting that there are more active ECs in AT [19]. It is not the tumor tissue itself, but the EC component of the tumor-induced stroma, that is responsible for recruiting EPCs. Our data also suggest that EPCs are recruited and home with high specificity to tumor tissues in an HCC mouse model without liver cirrhosis (unpublished data). Therefore, HCC coupled with cirrhosis provides a microenvironment that encourages the homing of EPCs.

In this study, the expression of angiogenic factors such as VEGF-A, bFGF, TGF-β, MCP-1, TSP-1, MMP-9, TIMP-2, and endostatin in AT was significantly higher than that in normal liver and HCC tissues. Many studies also report higher expression of proangiogenic factors such as VEGF-A [27-29], hepatic growth factor (HGF) [30], HIF-1α [19], and inducible nitric oxide [31] in the surrounding liver than in the tumor tissues. Moreover, increased levels of macrophage colony-stimulating factor and increased numbers of macrophages were found in peri-tumoral liver tissues than in the tumor tissue itself [32,33]. Of note, inhibitors of angiogenesis were also up-regulated along with the activators. Consistent with this, our previous study showed that the blood vessel density was higher in peri-tumoral tissues than in tumors [19]. Therefore, angiogenesis occurring in HCC with liver cirrhosis was different from that in other tumors. Most angiogenic factors showed higher expression levels in non-tumor tissues, which might be a reflection of both cirrhosis and a "field effect" relevant to the tumor. Taken together these studies suggest that the peri-tumoral tissues in HCC are an area of active angiogenesis.

However, the mechanism by which angiogenic factors are over-expressed in the peri-tumoral liver tissue remains unclear. HCC is a cancer associated, in most cases, with chronic liver diseases such as chronic viral hepatitis and cirrhosis, especially in Southeast Asia. The nonmalignant liver itself undergoes a precancerous change resulting in increased angiogenesis. During liver cirrhosis, fibrogenesis results in intrahepatic shunts and a barrier between the sinusoids and hepatocytes [34] generating areas of hypoxia. Our study indicates that the expression of HIF-1α, COX-2 and NOS-2 (which regulate the expression of many pro- and anti-angiogenic factors) is elevated in AT compared with TF and TT. This suggests that the distribution and expression of these angiogenic factors results from liver cirrhosis.

In the present study, we found that the high expression of angiogenic factors in the peri-tumoral liver tissue, but not in tumor tissue, was consistent with increasing microvessel density and EPC numbers. In addition, it is known that the MMPs and TIMPs upregulated in AT may be involved in the progression of HCC [35]. Therefore, the peri-tumoral tissues in HCC are an area of active angiogenesis, which may provide a microenvironment conducive for intrahepatic metastasis and tumor recurrence after resection. This is an important, but often neglected, issue. Together with these results, the present study implies that post-operative adjuvant therapies should target not only the residual tumor cells, but also the residual cirrhotic liver, which provides the microenvironment for intrahepatic metastasis and tumor recurrence. We propose that the peri-tumoral microenvironment is very important to understanding the mechanism of intrahepatic metastasis of HCC and in shaping the post-operative strategy for prevention of recurrence after hepatectomy.

## Conclusions

The present study shows that angiogenic factors expressed by peri-tumoral cells are responsible for the recruitment of EPCs and that the particular pathology found in HCC with liver cirrhosis may play an important role in the distribution, origin, and differentiation of EPCs. The results also highlight the fact that the peri-tumoral microenvironment may play an important role in tumor recurrence and metastasis after hepatectomy. Therefore, EPCs may be potential new targets or vectors for adjuvant therapy.

## Competing interests

The authors declare that they have no competing interests.

## Authors' contributions

DCY participated in the study design, carried out real-time PCR and Western blotting analysis and wrote the paper. JC participated in the study design, IHC and tissue array analysis, and wrote the paper. XTS participated in the study design, IHC and tissue array analysis. LYZ collected all the specimens and built the clinical database, and carried out IHC analysis. CPJ participated in the study design and helped to draft the manuscript. YTD conceived the study, participated in its design, and give final approval of the version to be published. All authors have read and approved the final manuscript.

## Pre-publication history

The pre-publication history for this paper can be accessed here:

http://www.biomedcentral.com/1471-2407/10/435/prepub

## Supplementary Material

Additional file 1 **Dynamics changes of circulating EPCs during liver cirrhosis**. The percent of c-Kit and Flk-1, Sca-1 and c-Kit, Sca-1 and Flk-1 positive cells was compared at 2, 4, 8 and 12 weeks versus 0 weeks (n = 5). *p < 0.05, **p < 0.01, c-Kit and Flk-1 positive cells; ^#^*p *< 0.05, ^##^*p *< 0.01, Sca-1 and c-Kit positive cells; $ *p *< 0.05; $$ *p *< 0.01, Sca-1 and Flk-1 positive cells. Click here for file

Additional file 2 **EPCs labeled with CM-Dil were validated**. Representative sections showed that EPCs were validated with FACS before (A, 2%) and after (B, 96.5%) being labeled with CM-Dil. Click here for file

Additional file 3 **Immunohistochemical staining of fibronectin in adjacent non-tumor tissues and tumor tissues**. Representative sections showing immunohistochemical staining of fibronectin in adjacent non-tumor tissues (A) and tumor tissues (B) (n = 30). The signals were detected by DAB staining. Magnification: ×200. Click here for file

Additional file 4 **Immunohistochemical staining of VCAM-1 in normal liver, adjacent non-tumor tissues and tumor tissues**. Representative sections showing immunohistochemical staining of VCAM-1 in normal liver (A), adjacent non-tumor tissues (B) and tumor tissues (C) (n = 30). The signals were detected by DAB staining. Magnification: ×200. Click here for file
