# Supplementary figures and images for: Mechanism of endothelial progenitor cell recruitment into neo-vessels in adjacent non-tumor tissues in hepatocellular carcinoma
Source: BMC Cancer. 2010 Aug 17;10:435. doi: 10.1186/1471-2407-10-435 (PMC2936325; doi:10.1186/1471-2407-10-435)

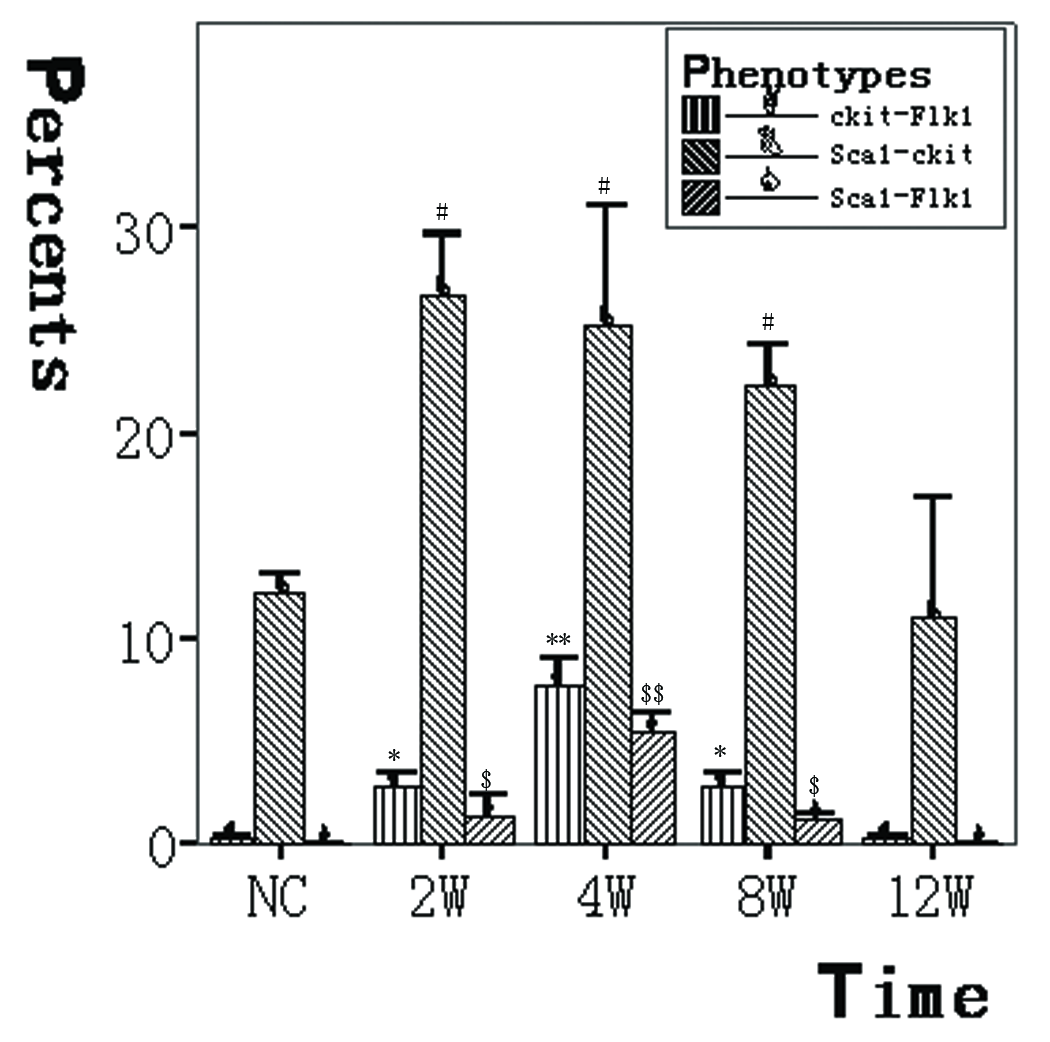

Supplement: Additional file 1 — Dynamics changes of circulating EPCs during liver cirrhosis. The percent of c-Kit and Flk-1, Sca-1 and c-Kit, Sca-1 and Flk-1 positive cells was compared at 2, 4, 8 and 12 weeks versus 0 weeks (n = 5). *p < 0.05, **p < 0.01, c-Kit and Flk-1 positive cells; #p < 0.05, ##p < 0.01, Sca-1 and c-Kit positive cells; $ p < 0.05; $$ p < 0.01, Sca-1 and Flk-1 positive cells. [file 1471-2407-10-435-S1.TIFF]

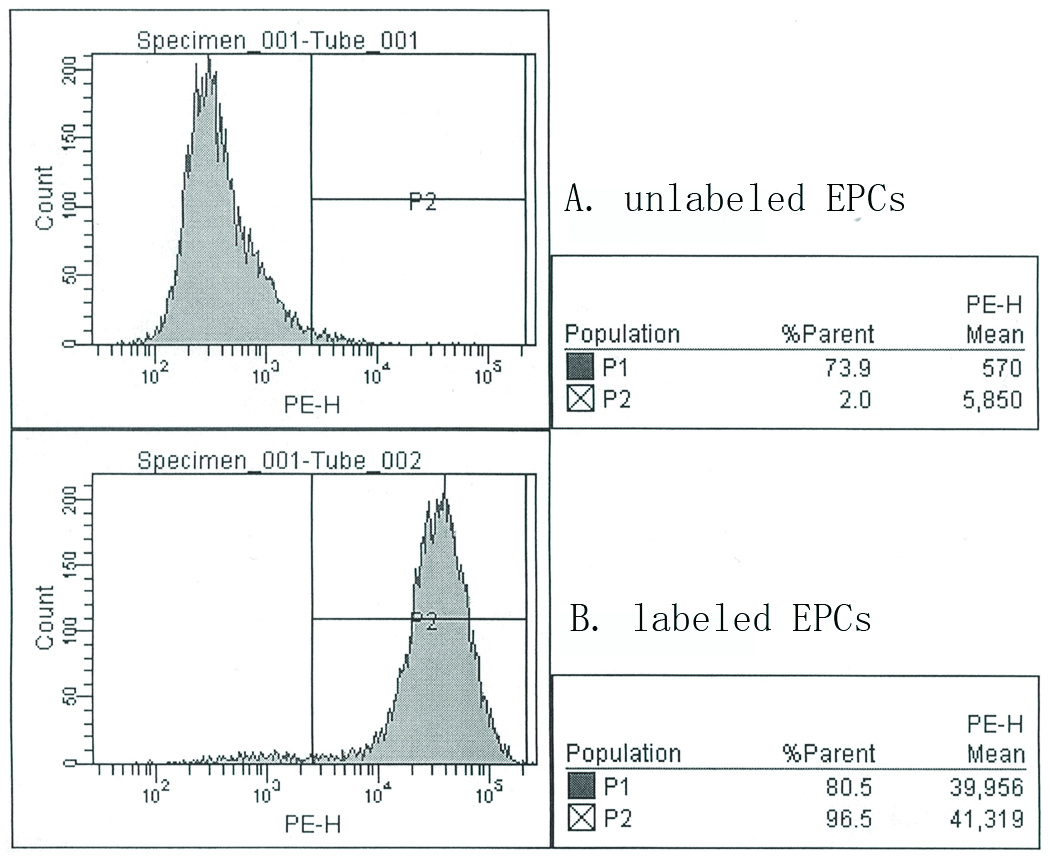

Supplement: Additional file 2 — EPCs labeled with CM-Dil were validated. Representative sections showed that EPCs were validated with FACS before (A, 2%) and after (B, 96.5%) being labeled with CM-Dil. [file 1471-2407-10-435-S2.TIFF]

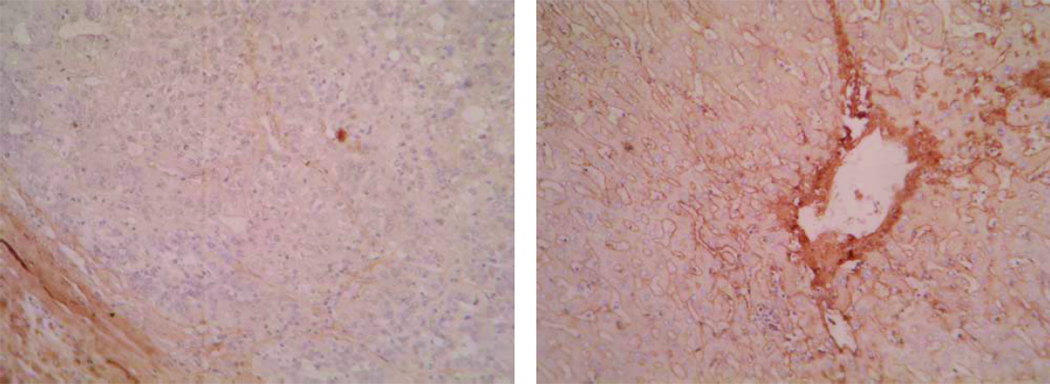

Supplement: Additional file 3 — Immunohistochemical staining of fibronectin in adjacent non-tumor tissues and tumor tissues. Representative sections showing immunohistochemical staining of fibronectin in adjacent non-tumor tissues (A) and tumor tissues (B) (n = 30). The signals were detected by DAB staining. Magnification: ×200. [file 1471-2407-10-435-S3.TIFF]

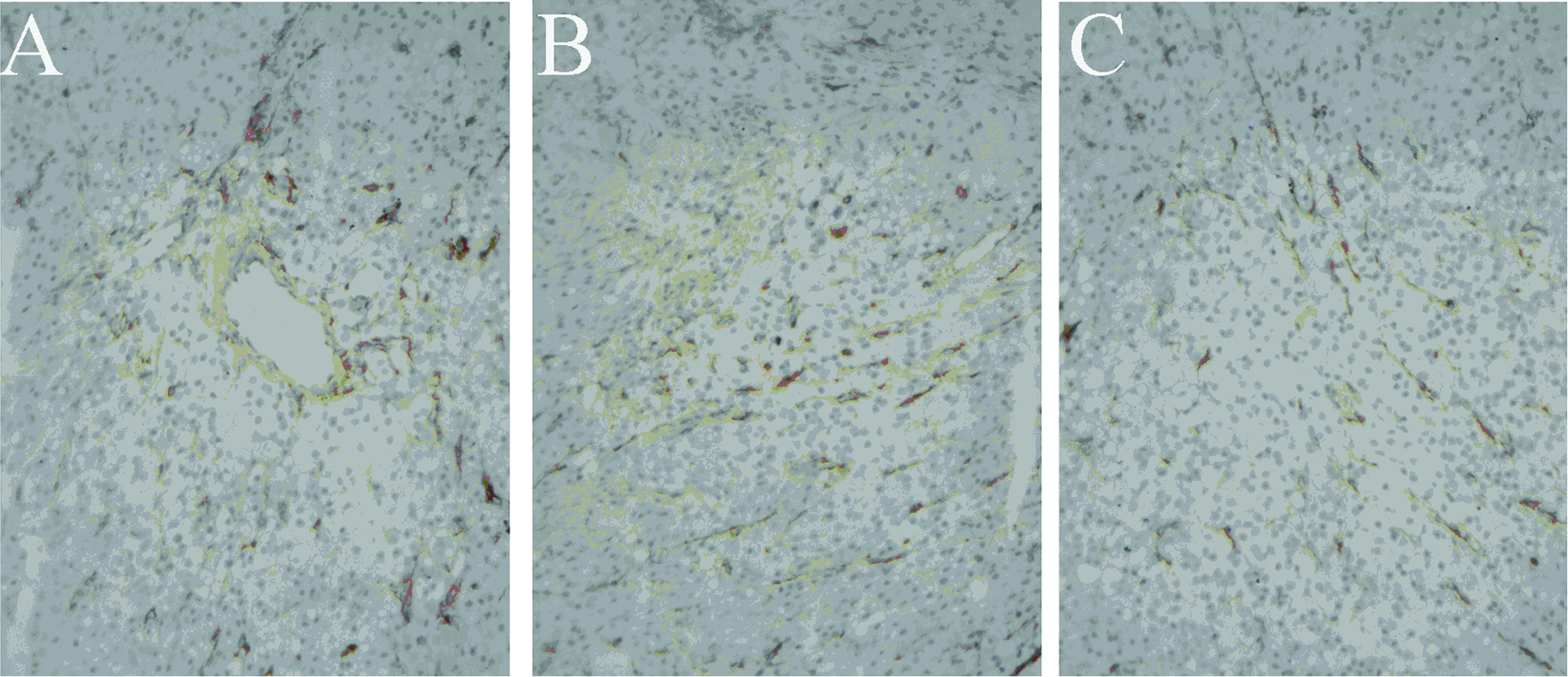

Supplement: Additional file 4 — Immunohistochemical staining of VCAM-1 in normal liver, adjacent non-tumor tissues and tumor tissues. Representative sections showing immunohistochemical staining of VCAM-1 in normal liver (A), adjacent non-tumor tissues (B) and tumor tissues (C) (n = 30). The signals were detected by DAB staining. Magnification: ×200. [file 1471-2407-10-435-S4.TIFF]
